# Solving patients with rare diseases through programmatic reanalysis of genome-phenome data

**DOI:** 10.1038/s41431-021-00852-7

**Published:** 2021-06-01

**Authors:** Leslie Matalonga, Carles Hernández-Ferrer, Davide Piscia, Solve-RD SNV-indel working group, Enzo Cohen, Isabel Cuesta, Daniel Danis, Anne-Sophie Denommé-Pichon, Yannis Duffourd, Christian Gilissen, Mridul Johari, Steven Laurie, Shuang Li, Leslie Matalonga, Isabelle Nelson, Sophia Peters, Ida Paramonov, Sivakumar Prasanth, Peter Robinson, Karolis Sablauskas, Marco Savarese, Wouter Steyaert, Joeri K. van der Velde, Antonio Vitobello, Rebecca Schüle, Matthis Synofzik, Ana Töpf, Lisenka E. L. M. Vissers, Richarda de Voer, Solve-RD DITF-GENTURIS, Stefan Aretz, Gabriel Capella, Richarda M. de Voer, Gareth Evans, Jose Garcia Pelaez, Elke Holinski-Feder, Nicoline Hoogerbrugge, Andreas Laner, Carla Oliveira, Andreas Rump, Evelin Schröck, Anna Katharina Sommer, Verena Steinke-Lange, Iris te Paske, Marc Tischkowitz, Laura Valle, Solve-RD DITF-ITHACA, Siddharth Banka, Elisa Benetti, Giorgio Casari, Andrea Ciolfi, Jill Clayton-Smith, Bruno Dallapiccola, Elke de Boer, Anne-Sophie Denommé-Pichon, Kornelia Ellwanger, Laurence Faivre, Holm Graessner, Tobias B. Haack, Anna Hammarsjö, Marketa Havlovicova, Alexander Hoischen, Anne Hugon, Adam Jackson, Tjitske Kleefstra, Anna Lindstrand, Estrella López-Martín, Milan Macek, Manuela Morleo, Vicenzo Nigro, Ann Nordgren, Maria Pettersson, Michele Pinelli, Simone Pizzi, Manuel Posada, Francesca Clementina Radio, Alessandra Renieri, Caroline Rooryck, Lukas Ryba, Martin Schwarz, Marco Tartaglia, Christel Thauvin, Annalaura Torella, Aurélien Trimouille, Alain Verloes, Lisenka Vissers, Antonio Vitobello, Pavel Votypka, Klea Vyshka, Birte Zurek, Solve-RD DITF-euroNMD, Jonathan Baets, Danique Beijer, Gisèle Bonne, Enzo Cohen, Judith Cossins, Teresinha Evangelista, Alessandra Ferlini, Peter Hackman, Michael G. Hanna, Rita Horvath, Henry Houlden, Mridul Johari, Jarred Lau, Hanns Lochmüller, William L. Macken, Francesco Musacchia, Andres Nascimento, Daniel Natera-de Benito, Vincenzo Nigro, Giulio Piluso, Veronica Pini, Robert D. S. Pitceathly, Kiran Polavarapu, Pedro M. Rodriguez Cruz, Anna Sarkozy, Marco Savarese, Rita Selvatici, Rachel Thompson, Bjarne Udd, Liedewei Van de Vondel, Jana Vandrovcova, Irina Zaharieva, Solve-RD DITF-RND, Jonathan Baets, Peter Balicza, Patrick Chinnery, Alexandra Dürr, Tobias Haack, Holger Hengel, Henry Houlden, Erik-Jan Kamsteeg, Christoph Kamsteeg, Katja Lohmann, Alfons Macaya, Anna Marcé-Grau, Ales Maver, Judit Molnar, Alexander Münchau, Borut Peterlin, Olaf Riess, Ludger Schöls, Rebecca Schüle-Freyer, Giovanni Stevanin, Matthis Synofzik, Vincent Timmerman, Bart van de Warrenburg, Nienke van Os, Melanie Wayand, Carlo Wilke, Raul Tonda, Steven Laurie, Marcos Fernandez-Callejo, Daniel Picó, Carles Garcia-Linares, Anastasios Papakonstantinou, Alberto Corvó, Ricky Joshi, Hector Diez, Ivo Gut, Alexander Hoischen, Holm Graessner, Sergi Beltran, the Solve-RD Consortia, Tobias B. Haack, Holm Graessner, Birte Zurek, Kornelia Ellwanger, Stephan Ossowski, German Demidov, Marc Sturm, Julia M. Schulze-Hentrich, Rebecca Schüle, Christoph Kessler, Melanie Wayand, Ludger Schöls, Holger Hengel, Peter Heutink, Han Brunner, Hans Scheffer, Nicoline Hoogerbrugge, Peter A. C. ’t Hoen, Wouter Steyaert, Karolis Sablauskas, Erik-Jan Kamsteeg, Bart van de Warrenburg, Iris te Paske, Erik Janssen, Marloes Steehouwer, Burcu Yaldiz, Anthony J. Brookes, Colin Veal, Spencer Gibson, Marc Wadsley, Mehdi Mehtarizadeh, Umar Riaz, Greg Warren, Farid Yavari Dizjikan, Thomas Shorter, Volker Straub, Chiara Marini Bettolo, Sabine Specht, Jill Clayton-Smith, Siddharth Banka, Elizabeth Alexander, Adam Jackson, Laurence Faivre, Christel Thauvin, Yannis Duffourd, Emilie Tisserant, Ange-Line Bruel, Christine Peyron, Aurore Pélissier, Sergi Beltran, Ivo Glynne Gut, Steven Laurie, Davide Piscia, Leslie Matalonga, Anastasios Papakonstantinou, Gemma Bullich, Alberto Corvo, Carles Garcia, Marcos Fernandez-Callejo, Carles Hernández, Daniel Picó, Ida Paramonov, Hanns Lochmüller, Gulcin Gumus, Virginie Bros-Facer, Ana Rath, Marc Hanauer, Annie Olry, David Lagorce, Svitlana Havrylenko, Katia Izem, Fanny Rigour, Alexandra Durr, Claire-Sophie Davoine, Léna Guillot-Noel, Anna Heinzmann, Giulia Coarelli, Gisèle Bonne, Teresinha Evangelista, Valérie Allamand, Isabelle Nelson, Rabah Ben Yaou, Corinne Metay, Bruno Eymard, Enzo Cohen, Antonio Atalaia, Tanya Stojkovic, Milan Macek, Marek Turnovec, Dana Thomasová, Radka Pourová Kremliková, Vera Franková, Markéta Havlovicová, Vlastimil Kremlik, Helen Parkinson, Thomas Keane, Dylan Spalding, Alexander Senf, Daniel Danis, Glenn Robert, Alessia Costa, Christine Patch, Mike Hanna, Henry Houlden, Mary Reilly, Jana Vandrovcova, Francesco Muntoni, Anna Sarkozy, Vincent Timmerman, Jonathan Baets, Liedewei Van de Vondel, Danique Beijer, Peter de Jonghe, Sandro Banfi, Annalaura Torella, Alessandra Ferlini, Rita Selvatici, Rachele Rossi, Marcella Neri, Stefan Aretz, Isabel Spier, Sophia Peters, Carla Oliveira, Jose Garcia Pelaez, Ana Rita Matos, Celina São José, Marta Ferreira, Irene Gullo, Susana Fernandes, Luzia Garrido, Pedro Ferreira, Fátima Carneiro, Morris A. Swertz, Lennart Johansson, Gerben van der Vries, Pieter B. Neerincx, Dieuwke Roelofs-Prins, Sebastian Köhler, Alison Metcalfe, Caroline Rooryck, Aurelien Trimouille, Raffaele Castello, Manuela Morleo, Alessandra Varavallo, Manuel Posada De la Paz, Eva Bermejo Sánchez, Estrella López Martín, Beatriz Martínez Delgado, F. Javier Alonso García de la Rosa, Francesca Clementina Radio, Marco Tartaglia, Alessandra Renieri, Elisa Benetti, Peter Balicza, Maria Judit Molnar, Ales Maver, Borut Peterlin, Alexander Münchau, Katja Lohmann, Rebecca Herzog, Martje Pauly, Alfons Macaya, Anna Marcé-Grau, Andres Nascimiento Osorio, Daniel Natera de Benito, Hanns Lochmüller, Rachel Thompson, Kiran Polavarapu, David Beeson, Judith Cossins, Pedro M. Rodriguez Cruz, Peter Hackman, Mridul Johari, Marco Savarese, Bjarne Udd, Rita Horvath, Gabriel Capella, Laura Valle, Elke Holinski-Feder, Andreas Laner, Verena Steinke-Lange, Evelin Schröck, Andreas Rump

**Affiliations:** 1 CNAG-CRG, Centre for Genomic Regulation (CRG), The Barcelona Institute of Science and Technology, Baldiri Reixac 4, Barcelona, Spain; 2 Department of Neurodegeneration, Hertie Institute for Clinical Brain Research (HIH), University of Tübingen, Tübingen, Germany; 3 German Center for Neurodegenerative Diseases (DZNE), Tübingen, Germany; 4 John Walton Muscular Dystrophy Research Centre, Translational and Clinical Research Institute, Newcastle University and Newcastle Hospitals NHS Foundation Trust, Newcastle upon Tyne, UK; 5 Department of Human Genetics, Radboud University Medical Center, Nijmegen, The Netherlands; 6 Donders Institute for Brain, Cognition and Behaviour, Radboud University Medical Center, Nijmegen, The Netherlands; 7 Radboud Institute for Molecular Life Sciences, Nijmegen, The Netherlands; 8 Department of Internal Medicine and Radboud Center for Infectious Diseases (RCI), Radboud University Medical Center, Nijmegen, The Netherlands; 9 Institute of Medical Genetics and Applied Genomics, University of Tübingen, Tübingen, Germany; 10 European Reference Network for Rare Neurological Diseases, Tübingen, Germany; 11 Universitat Pompeu Fabra (UPF), Barcelona, Spain; 12 Departament de Genètica, Microbiologia i Estadística, Facultat de Biologia, Universitat de Barcelona (UB), Barcelona, Spain; 13 Sorbonne Université, INSERM UMRS_974, Center of Research in Myology, Paris, France; 14 Institute of Rare Diseases Research, Spanish Undiagnosed Rare Diseases Cases Program (SpainUDP) & Undiagnosed Diseases Network International (UDNI), Instituto de Salud Carlos III, Madrid, Spain; 15 Jackson Laboratory for Genomic Medicine, Farmington, CT USA; 16 Inserm - University of Burgundy-Franche Comté, UMR1231 GAD Dijon, France; 17 Dijon University Hospital, Genetics Department, Dijon, France; 18 Dijon University Hospital, FHU-TRANSLAD, Dijon, France; 19 Folkhälsan Research Center, University of Helsinki, Helsinki, Finland; 20 Department of Genetics, Genomics Coordination Center, University Medical Center Groningen, University of Groningen, Groningen, The Netherlands; 21 Institute of Human Genetics, University of Bonn, Bonn, Germany; 22 MRC Centre for Neuromuscular Diseases and National Hospital for Neurology and Neurosurgery, UCL Queen Square Institute of Neurology, London, UK; 23 Center for Hereditary Tumor Syndromes, University Hospital Bonn, Bonn, Germany; 24 Division of Evolution and Genomic Sciences, School of Biological Sciences, Faculty of Biology, Medicine and Health, University of Manchester, Manchester, UK; 25 i3S - Instituto de Investigação e Inovação em Saúde, Universidade do Porto, Porto, Portugal; 26 IPATIMUP - Institute of Molecular Pathology and Immunology of the University of Porto, Porto, Portugal; 27 University of Munich, Munich, Germany; 28 Departament of Pathology, Faculty of Medicine, University of Porto, Porto, Portugal; 29 Universty of Dresden, Dresden, Germany; 30 Department of Medical Genetics, National Institute for Health Research Cambridge Biomedical Research Centre, University of Cambridge, Cambridge, UK; 31 Instituto de Investigación de Bellvitge, Hospitalet de Llobregat, Llobregat, Spain; 32 Manchester Centre for Genomic Medicine, St Mary’s Hospital, Manchester University Hospitals NHS Foundation Trust, Health Innovation Manchester, Manchester, UK; 33 Manchester Centre for Genomic Medicine, Division of Evolution and Genomic Sciences, School of Biological Sciences, Faculty of Biology, Medicine and Health, University of Manchester, Manchester, UK; 34 Med Biotech Hub and Competence Center, Department of Medical Biotechnologies, University of Siena, Siena, Italy; 35 Dipartimento di Medicina di Precisione, Università degli Studi della Campania “Luigi Vanvitelli,”, Napoli, Italy; 36 Telethon Institute of Genetics and Medicine, Pozzuoli, Italy; 37 Genetics and Rare Diseases Research Division, Ospedale Pediatrico Bambino Gesù, IRCCS, Rome, Italy; 38 Laboratoire de Génétique Moléculaire, Service de Génétique Médicale, CHU Bordeaux – Hôpital Pellegrin, Place Amélie Raba Léon, Bordeaux Cedex, France; 39 Centre for Rare Diseases, University of Tübingen, Tübingen, Germany; 40 Dijon University Hospital, Genetics Department and Centres of Reference for Development disorders and intellectual disabilities, FHU TRANSLAD and GIMI Institute, Dijon, France; 41 Karolinska Institutet, Solna, Sweden; 42 Department of Biology and Medical Genetics, Charles University Prague-2nd Faculty of Medicine and University Hospital Motol, Prague, Czech Republic; 43 Department of Genetics, Assistance Publique-Hôpitaux de Paris - Université de Paris, Robert DEBRE University Hospital, 48 bd SERURIER, Paris, France; 44 Ospedale Pediatrico Bambino Gesù, Rome, Italy; 45 Medical Genetics, University of Siena, Siena, Italy; 46 Genetica Medica, Azienda Ospedaliero-Universitaria Senese, Siena, Italy; 47 Université Bordeaux, MRGM INSERM U1211, CHU de Bordeaux, Service de Génétique Médicale, Bordeaux, France; 48 INSERM UMR 1141 “NeuroDiderot”, Hôpital R DEBRE, Paris, France; 49 Translational Neurosciences, Faculty of Medicine and Health Sciences, UAntwerpen, Antwerp, Belgium; 50 Laboratory of Neuromuscular Pathology, Institute Born-Bunge, University of Antwerp, Antwerpen, Belgium; 51 Neuromuscular Reference Centre, Department of Neurology, Antwerp University Hospital, Antwerpen, Belgium; 52 Neuromuscular Disorders Group, NDCN, Weatherall Institute of Molecular Medicine, John Radcliffe Hospital, Oxford, UK; 53 Unit of Medical Genetics, Department of Medical Sciences, University of Ferrara, Ferrara, Italy; 54 Folkhälsan Research Center, University of Helsinki and Tampere Neuromuscular Center, Tampere, Finland; 55 Department of Neuromuscular Diseases, UCL Queen Square Institute of Neurology and The National Hospital for Neurology and Neurosurgery, London, UK; 56 University of Cambridge, England, UK; 57 Children’s Hospital of Eastern Ontario Research Institute, Ottawa, Canada; 58 Division of Neurology, Department of Medicine, The Ottawa Hospital, Ottawa, Canada; 59 Brain and Mind Research Institute, University of Ottawa, Ottawa, Canada; 60 Department of Neuropediatrics and Muscle Disorders, Medical Center – University of Freiburg, Faculty of Medicine, Freiburg, Germany; 61 Neuromuscular Unit, Neuropaediatrics Department, Institut de Recerca Pediàtrica Hospital Sant Joan de Déu, CIBERER, Barcelona, Spain; 62 Dubowitz Neuromuscular Centre, UCL Great Ormond Street Hospital, London, UK; 63 Nuffield Department of Clinical Neurosciences, University of Oxford, Oxford, UK; 64 Peripheral Neuropathy Research Group, University of Antwerp, Antwerp, Belgium; 65 Semelweis University Budapest, Budapest, Hungary; 66 Institut National de la Santé et de la Recherche Medicale (INSERM) U1127, Paris, France; 67 Centre National de la Recherche Scientifique, Unité Mixte de Recherche (UMR) 7225, Paris, France; 68 Unité Mixte de Recherche en Santé 1127, Université Pierre et Marie Curie (Paris 06), Sorbonne Universités, Paris, France; 69 University of Lübeck, Lübeck, Germany; 70 Hospital Vall d’Hebron, Barcelona, Spain; 71 University of Ljubljana, Ljubljana, Slovenia; 72 Institut du Cerveau-ICM, Paris, France; 73 Ecole Pratique des Hautes Etudes, Paris Sciences et Lettres Research University, Paris, France; 74 Peripheral Neuropathy Research Group, Department of Biomedical Sciences, University of Antwerp, Antwerp, Belgium; 75 Institute Born Bunge, Antwerp, Belgium; 76 Department of Neurology, Radboud University Medical Center, Nijmegen, The Netherlands; 77 Department of Clinical Genetics, Maastricht University Medical Centre, Maastricht, The Netherlands; 78 Center for Molecular and Biomolecular Informatics, Radboud University Medical Center, Nijmegen, The Netherlands; 79 Department of Genetics and Genome Biology, University of Leicester, Leicester, UK; 80 Dijon University Hospital, Centre of Reference for Rare Diseases: Development Disorders and Malformation Syndromes, Dijon, France; 81 Dijon University Hospital, GIMI institute, Dijon, France; 82 University of Burgundy-Franche Comté, Dijon Economics Laboratory, Dijon, France; 83 University of Burgundy-Franche Comté, FHU-TRANSLAD, Dijon, France; 84 EURORDIS-Rare Diseases Europe, Sant Antoni Maria Claret 167 - 08025, Barcelona, Spain; 85 EURORDIS-Rare Diseases Europe, Plateforme Maladies Rares, Paris, France; 86 INSERM, US14 - Orphanet, Plateforme Maladies Rares, Paris, France; 87 Centre de Référence de Neurogénétique, Hôpital de la Pitié-Salpêtrière, Assistance Publique-Hôpitaux de Paris (AP-HP), Paris, France; 88 Hôpital de la Pitié-Salpêtrière, Assistance Publique-Hôpitaux de Paris (AP-HP), Paris, France; 89 AP-HP, Centre de Référence de Pathologie Neuromusculaire Nord, Est, Ile-de-France, Institut de Myologie, G.H. Pitié-Salpêtrière, Paris, France; 90 Institut de Myologie, Equipe Bases de données, G.H. Pitié-Salpêtrière, Paris, France; 91 AP-HP, Unité Fonctionnelle de Cardiogénétique et Myogénétique Moléculaire et Cellulaire, G.H. Pitié-Salpêtrière, Paris, France; 92 European Bioinformatics Institute, European Molecular Biology Laboratory, Wellcome Genome Campus, Hinxton, Cambridge, UK; 93 Florence Nightingale Faculty of Nursing and Midwifery, King’s College, London, UK; 94 Genetic Counselling, Genomics England, Queen Mary University of London, Dawson Hall, EC1M 6BQ London, UK; 95 Department of Neuromuscular Diseases, UCL Queen Square Institute of Neurology, London, UK; 96 NIHR Great Ormond Street Hospital Biomedical Research Centre, London, UK; 97 Departament of Genetics, Faculty of Medicine, University of Porto, Porto, Portugal; 98 CHUSJ, Centro Hospitalar e Universitário de São João, Porto, Portugal; 99 Faculty of Sciences, University of Porto, Porto, Portugal; 100 NeuroCure Cluster of Excellence, Charité Universitätsklinikum, Charitéplatz 1, 10117 Berlin, Germany; 101 College of Health, Well-being and Life-Sciences, Sheffield Hallam University, Sheffield, UK; 102 Institute of Genomic Medicine and Rare Diseases, Semmelweis University, Budapest, Hungary; 103 Clinical Institute of Genomic Medicine, University Medical Centre Ljubljana, Ljubljana, Slovenia; 104 Institute of Neurogenetics, University of Lübeck, Lübeck, Germany; 105 Neurology Research Group, Vall d’Hebron Research Institute, Universitat Autònoma de Barcelona, Barcelona, Spain; 106 Neuromuscular Disorders Unit, Department of Pediatric Neurology, Hospital Sant Joan de Déu, Barcelona, Spain; 107 Centro Nacional de Análisis Genómico (CNAG-CRG), Center for Genomic Regulation, Barcelona Institute of Science and Technology (BIST), Barcelona, Spain; 108 Children’s Hospital of Eastern Ontario Research Institute, University of Ottawa, Ottawa, ON Canada; 109 Folkhälsan Research Centre and Medicum, University of Helsinki, Helsinki, Finland; 110 Tampere Neuromuscular Center, Tampere, Finland; 111 Vasa Central Hospital, Vaasa, Finland; 112 Department of Clinical Neurosciences, University of Cambridge, Cambridge, UK; 113 Bellvitge Biomedical Research Institute (IDIBELL), Barcelona, Spain; 114 Medical Genetics Center (MGZ), Munich, Germany; 115 Institute for Clinical Genetics, Faculty of Medicine Carl Gustav Carus, Technical University Dresden, Dresden, Germany; 116 Center for Personalized Oncology, University Hospital Carl Gustav Carus, Technical University Dresden, Dresden, Germany

**Keywords:** Diseases, Genetic testing, Genome informatics, Genomic analysis

## Abstract

Reanalysis of inconclusive exome/genome sequencing data increases the diagnosis yield of patients with rare diseases. However, the cost and efforts required for reanalysis prevent its routine implementation in research and clinical environments. The Solve-RD project aims to reveal the molecular causes underlying undiagnosed rare diseases. One of the goals is to implement innovative approaches to reanalyse the exomes and genomes from thousands of well-studied undiagnosed cases. The raw genomic data is submitted to Solve-RD through the RD-Connect Genome-Phenome Analysis Platform (GPAP) together with standardised phenotypic and pedigree data. We have developed a programmatic workflow to reanalyse genome-phenome data. It uses the RD-Connect GPAP’s Application Programming Interface (API) and relies on the big-data technologies upon which the system is built. We have applied the workflow to prioritise rare known pathogenic variants from 4411 undiagnosed cases. The queries returned an average of 1.45 variants per case, which first were evaluated in bulk by a panel of disease experts and afterwards specifically by the submitter of each case. A total of 120 index cases (21.2% of prioritised cases, 2.7% of all exome/genome-negative samples) have already been solved, with others being under investigation. The implementation of solutions as the one described here provide the technical framework to enable periodic case-level data re-evaluation in clinical settings, as recommended by the American College of Medical Genetics.

## Introduction

According to some estimations, around 350 million people worldwide may suffer from one of at least 7000 existing rare diseases (RDs) [1]. As 80% of RDs are thought to have a genetic origin [2, 3], the identification and characterisation of the molecular basis underlying these disorders is crucial for the establishment of a specific diagnosis and the subsequent identification of an optimal therapeutic approach.

The next generation sequencing (NGS) era has enabled cost-effective sequencing of RD patients’ exome or genome, bringing these approaches into diagnostics [4]. However, the identification and interpretation of disease-causing variants remains challenging. Indeed, the reported diagnostic yield for exome sequencing of RD patients with suspected monogenic disorders is around 20–60% depending on the type of disorder [5–7]. Undiagnosed cases can be re-approached by generating new genetic data using other techniques with more sensitivity than NGS for certain types of variants (e.g. arrays for large deletions or duplications) or re-sequencing the samples using other library strategies and sequencing protocols (e.g. whole genome sequencing, deep exon sequencing, a different exon capture kit, etc.).

Nevertheless, a negative result from NGS does not mean that the disease aetiology lies outside of the data already produced. In some cases, the variant is missed due to the bioinformatics analysis or incomplete phenotypic or family information. In other cases, the variant is not pinpointed because, at the time, the impact cannot be adequately assessed and/or the gene has not been yet associated with a certain function. However, technical developments and scientific understanding are constantly expanding, with new gene-disease associations increasing at an average rate of 250 per year (based on OMIM) and 9200 variant–disease associations being curated each year (based on HGMD) [8]. As a result, periodic data reanalysis and/or re-evaluation increases the diagnostic yield up to 10–12% [9–11], and the American College of Medical Genomics (ACMG) recommends variant-level re-evaluation and case-level reanalysis every 2 years [12].

While the scientific community extensively agrees on the benefits of periodic data reanalysis for RD patients, frequent re-evaluation of exomes/genomes is challenging in practice. The time-consuming effort required to identify the clinical record and re-assess segregated and unstructured genome-phenome data, together with the non-scalability of current solutions to reanalyse exponentially-growing datasets over time, preclude its implementation in research and clinical practice. Indeed, most clinical centres still do not include any re-evaluation approach in their routinely workflow as the benefit of identifying a new diagnosis is hardly unbalanced compared with the cost and efforts required for reanalysis. Therefore, innovative bioinformatics solutions are crucial to overcome some of these issues and facilitate iterative re-evaluation processes [11].

Solve-RD (http://solve-rd.eu/) aims to reveal the molecular cause underlying undiagnosed RDs [13]. One of the main goals of the project is to comprehensively reanalyse more than 19,000 phenotypically well characterised exome/genome negative datasets from unsolved patients with RDs submitted by European Reference Networks (ERNs). Besides the genomic data, the datasets include the phenotypic and pedigree information according to the RD-REAL (Rare Disease - REAnalysis Logistics) minimum information recommended for reanalysis [13]. All the existing RD-REAL datasets and the new ones generated by the project are being submitted to the RD-Connect Genome-Phenome Analysis Platform (GPAP, https://platform.rd-connect.eu/) as an entry point to the Solve-RD project.

The RD-Connect GPAP is an online platform that facilitates genome-phenome data analysis for RD diagnosis and gene discovery. Since datasets are submitted by many clinical researchers and are generated in different clinical centres and genomic facilities, the data are quite diverse at the source. To harmonise the information across all patients and relatives, the GPAP enables submission of pseudonymised phenotypic and clinical data using ontologies and standards such as the Human Phenotype Ontology (HPO) [14], the Orphanet Rare Disease Ontology (ORDO) [1], and the Online Mendelian Inheritance in Man database (OMIM) [2]. All the genomic data is processed through the same standardised pipeline [15] before being annotated and stored in an Elasticsearch database, which provides low-latency queries to enable fast access and ensure scalability.

Herein we describe a novel method that enables an automated, flexible, fast and iterative re-evaluation of thousands of genomic datasets using a programmatic access to the RD-Connect GPAP and we illustrate the utility of this procedure by reanalysing 4411 exome/genome negative index cases from the Solve-RD project. This approach has enabled the diagnosis of the first 120 cases within Solve-RD.

## Patient and methods

### Subjects

This study includes phenotypic and genomic data from 4703 affected individuals (4411 families) and 3690 unaffected relatives submitted to the RD-Connect GPAP as part of the Solve-RD project (http://solve-rd.eu/) [13] by four European Reference networks (the European Reference Networks for Rare Neurological Diseases (ERN-RND), Neuromuscular Diseases (ERN Euro NMD), Intellectual Disability and Congenital Malformations (ERN ITHACA) and Genetic Tumor Risk Syndromes (ERN GENTURIS), https://ec.europa.eu/health/ern_en), as well as two Undiagnosed Disease Programs (UDP Italy and UDP Spain). Clinical information was collated in a standard format using the HPO [14] for symptoms and the ORDO [1] for Clinical disorders. Each patient entry was associated with its corresponding submitting group and linked to its corresponding ERN or UDP. The responsibility of checking the data is suitable for submission to the RD-Connect GPAP and Solve-RD lies within the data submitter as required by their Code of Conduct and Data Sharing Policy, respectively. In some cases, individuals had to be re-consented prior to data submission. This study adheres to the principles set out in the Declaration of Helsinki.

### Genomic data processing

4551 exome and 201 genome sequencing data (FastQ or BAM) derived from the 4703 affected individuals included in the Solve-RD freeze 1 dataset, were processed using the RD-Connect GPAP standardised analysis pipeline based upon GATK3.6 best practices and using the GRCh37 human reference, as described in ref. [15]. The resulting variants, including single nucleotide variants (SNVs), short insertions and deletions (InDels) and mtDNA variants (when captured) were annotated using VEP [16]. In addition, GnomAD [17], and ClinVar [18] were annotated with the latest versions available as for January 2020. Each dataset was associated with its corresponding phenotypic data and tagged with the name of the submitting ERN or UDP. Data are available to authorised users for analysis through the RD-Connect GPAP user interface (https://platform.rd-connect.eu/).

### Programmatic access to genome-phenome datasets

Annotated genomic data is indexed in a non-relational ElasticSearch database engine (https://github.com/elastic/elasticsearch, GitHub - elastic/elasticsearch) connected to a Hadoop environment (Apache Software Foundation, https://hadoop.apache.org). Phenotypic data is stored in a local phenotypic database. Both genomic and phenotypic data are made computationally accessible through Application Programming Interface (API) endpoints, allowing automated queries through an in-house python package. To ensure secure and GDPR (General Data Protection Regulation) compliant data access for authorised users, the python package integrates a keycloak user authentication and permission management (github.com/keycloak/keycloak, GitHub - keycloak).

The GPAP’s API enables programmatic and flexible data analysis by (i) applying any type of filtering parameters according to the GPAP variants annotation (e.g. population frequencies, protein impact and in silico predictors), (ii) integrating standardised phenotypic information from each index case to create unique on-the-fly gene list for each of the experiments, (iii) filtering by specific gene lists according to the type of disorder (curated by ERNs, remote access to PanelApp from Genomics England or genes from any local or public database of interest), (iv) restraining the query filtering by homozygous regions in consanguineous cases or by specific regions of interest (e.g. regulatory regions) and (v) include segregation analysis based on the suspected inheritance and data from patient relatives introduced in the system.

### Variant filtering parameters

**Fig. 1:**
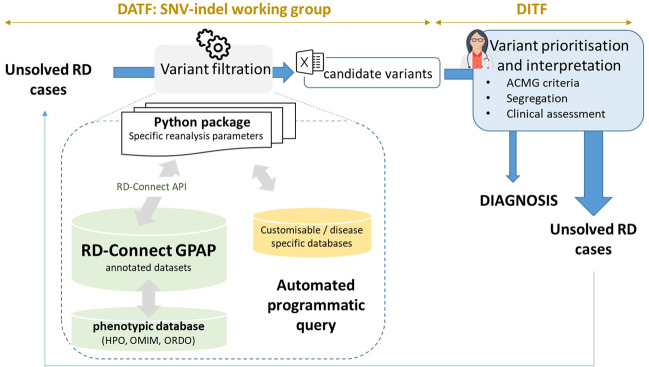
Programmatic reanalysis data workflow. Unsolved cases (RD-REAL datasets = phenotypic and genomic data) are submitted by Solve-RD members from the 4 core ERNs and the 2 UDPs participating in the project. Genomic data is processed through a standard analysis pipeline [15] and integrated with the phenotypic information in the RD-Connect GPAP. Analysis of the data using the programmatic approach described in this study is performed by the SNV-indel working group. The SNV-indel working group is one of the seven working groups established by the Solve-RD Data Analysis Task Force (DATF) to massively reanalyse data with different analytical approaches (e.g. CNV, somatic, meta-analysis, etc.) (http://solve-rd.eu/the-group/data-analysis-task-force/). The DATF involves data scientists and genomics experts from the project. Resulting candidate variants are submitted to the Data Interpretation Task Force (DITF), involving expert clinicians and geneticists for prioritisation and final interpretation. One DITF has been established for each of the core ERNs participating in the project (http://solve-rd.eu/the-group/data-interpretation-task-force-ditf/). DITF include or are in contact with case submitters to enable a final decision for a new patient diagnosis. Diagnosed cases are automatically updated in the system and the remaining unsolved cases are susceptible to re-enter a new round of analysis.

Variant filtering using the RD-Connect GPAP’s programmatic access described above was applied to identify candidate disease-causing SNVs and, InDels using the following parameters: [1] rare variants (observed population allele frequency <0.01 according to gnomAD and <0.02 according to the RD-Connect GPAP internal frequency), [2] specific gene list provided by the corresponding ERNs (euro-NMD, RND, ITHACA and GENTURIS) and [3] variant annotated as pathogenic or likely pathogenic for a specific disorder in ClinVar (v.13-01-2020). Apart from standard annotations (VEP), the resulting output file (one per ERN) was annotated with pseudonymised IDs, patient standardised phenotypic information (by extracting the corresponding HPOs and ORDO information entered in the system), candidate gene-disease associations (according to OMIM) [2], consanguinity reported and experimentally inferred (according to ref. [19]), gene constrain scores (pLI and o/e according to gnomAD v.2.1.1), ACMG computationally predicted clinical significance and criterias (using InterVar) [20] and when relevant, specific disease pathogenicity databases such as the VKGL database (https://www.vkgl.nl/nl/diagnostiek/vkgl-datashare-database) and the gene4denovo database [21]. The overall approach was designed by the Solve-RD SNV-indel working group from the Data Analysis Task Force (DATF) in collaboration with the corresponding disease expert groups [13] (Fig. 1).

### Variant prioritisation and data interpretation

Candidate variants from each case passing the filtering criteria are included in a single table to facilitate distribution across the Solve-RD network for evaluation and provision of feedback. The table is in MS Excel and has the same, or very similar, structure as the one provided by other Solve-RD DATF Working Groups for other type of genomic analyses. Solve-RD has organised ERNs clinical expertise in four dedicated Data Interpretation Task Forces (DITFs), one for each of the core ERNs. Results from the programmatic reanalysis performed were sent to the corresponding DITF members, a group of dedicated disease experts from the project who prioritised variants for further clinical assessment by data submitters (Fig. 1). Variant interpretation was then carried out in accordance with the criteria set by the ACMG guidelines [22] and the posterior ClinGen Sequence Variant Interpretation recommendations (https://www.clinicalgenome.org/working-groups/sequence-variant-interpretation/). The final feedback of variant pathogenicity for a specific clinical condition was determined by integrating patient assessment, variant evaluation and segregation, suspected inheritance, and clinical fit. Concerning family data available for segregation analyses, 28% of cases were submitted as trios (80% of them from ITHACA families), 68% were submitted as singletons (62% of them from RND) and 4% were from other family structures (Table 1).

**Table. 1:** Number of cases, family structures and identified variants by European Reference Networks participating in the study.

Type of disorder	Number of families /index cases	Trio	Singleton	Other family structure	Number of genes in the corresponding gene list	Number variants identified	Number of cases with identified variants	Number of variants prioritised	Number of cases with prioritised variants	Number of solved cases	Number of cases under evaluation	Number of cases with an heterozygous variant for an AR disorder identified	Number of unsolved cases
Intellectual disability	1472	1008 (68.4%)	436 (29.6%)	28 (2%)	1740	1618	980	193	158	62	5	15	76
Neuromuscular disorders	616	124 (20.1%)	433 (70.2%)	59 (9.5%)	594	278	223	278	228	22	13	21	172
Neurological disorders	2048	130 (6.3%)	1847 (90.1%)	71 (3.4%)	358	667	552	177	150	38	2	48	62
Tumor risk syndromes	275	0	273 (99.3%)	2 (0.7%)	229	30	30	30	30	3	0	3	24
TOTAL	4411	1262 (28%)	2989 (68%)	160 (4%)	NA	2593	1785	678	566	120	25	87	334

## Results

### Programmatic reanalysis workflow

To enable automated and reproducible analysis and reanalysis of the Solve-RD data, we have developed a python package to execute queries through the RD-Connect API in a secure manner (Fig. 1). The parameters must be indicated in a configuration file, allowing a flexible (re)analysis environment covering very high to very low filtering stringencies and integrating patient clinical information through the use of computer readable standards (HPOs, ORDO, and OMIM) (Fig. 1). Options available for filtering include all annotations and features integrated in the RD-Connect GPAP from standard annotations (e.g. internal and external population allele frequencies) to more advanced features integrating clinical information to create patient specific on-the-fly gene lists (e.g. gene lists based on the HPOs entered for the index case). At the time being, the approach can detect SNVs and small InDels, including canonical splicing mutations. Other type of variants such as copy number variants will be integrated in the GPAP for filtering in future releases. In the meantime, Solve-RD has a specific DATF Working Group performing CNV analyses. Whenever relevant, the CNV variants are combined with the SNV/InDel results outside of the GPAP.

The queries are executed sequentially on the selected cases, enabling a scalable and tailored approach. The GPAP currently contains variants from 12,335 exomes and 638 genomes, distributed across 30 ElasticSearch instances in 12 server nodes (each with 2 octa-cores at 2.60 GHz, 256GB RAM and SSD disks). On these settings, each query requires 30 s per experiment on average.

The resulting variants are distributed to the respective DITF for variant prioritisation and interpretation (Fig. 1). After evaluation, the causative variants are tagged in the RD-Connect GPAP through the API or the graphical user-friendly interface. Unsolved cases may enter a new round of interpretation with a different combination of parameters and filters. New rounds of analysis are designed in collaboration with each of the DITF. Current approaches concern, for example, the identification of homozygous variants in homozygous stretches greater than 1 Mb for consanguineous cases or the identification of variants in known regulatory regions for specific patient cohorts (e.g. congenital myasthenic syndrome). Furthermore, other types of analyses are being done within Solve-RD, as indicated in ref. [13].

### Application of the programmatic workflow for the reanalysis of undiagnosed rare disease patients

Bioinformatics reanalysis and the programmatic evaluation workflow were applied to all affected cases in the Solve-RD freeze 1 dataset [13]. In total, 4411 undiagnosed cases with heterogeneous genetic disorders were included: 1472 index cases referred as Intellectual disability (ERN-ITHACA), 2048 as Rare Neurological Disorder (ERN-RND), 616 as Neuromuscular Disorders (ERN-euroNMD), and 275 as Tumor Risk Syndromes (ERN-GENTURIS). Among the whole dataset, 55.7% of the cases were males and 44.3% females.

To minimise the interpretation burden for the DITFs, the first round of analysis was designed with very stringent parameters to allow the identification of clear candidates (”low-hanging fruit”) with known disease causality (Table 1, Fig. 1). All candidate variants were reported as “pathogenic” or “likely pathogenic” in ClinVar. Pathogenic variants are defined (based on the ACMG) as variants that directly contribute to the development of a disorder in a specific dosage sensitivity. The latter meaning that some pathogenic variants may not be fully penetrant or in the case of recessive or X-linked conditions, a single pathogenic variant may not be sufficient to cause disease on its own.

**Fig. 2:**
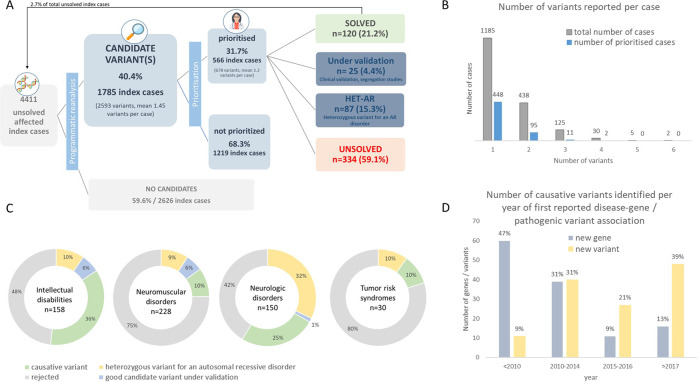
Results of reanalysis of undiagnosed RD cases to identify known disease-causing variants. **A** Filtration, prioritisation and interpretation workflow (numbers refer to index cases). **B** Number of variants per case submitted to DITFs for prioritisation and resulting number of variants submitted for interpretation. **C** Variants interpretation results from prioritised cases per type of disorder (numbers refer to variants). **D** Number of causative variants identified according to the year the corresponding gene (grey) or variant (yellow) was first described in the literature as disease-causing (according to OMIM) or pathogenic (according to ClinVar).

Total computational time for this analysis (including filtering and additional annotation steps for all 4411 experiments) was of 36 h and 45 min. The analysis yielded a total of 2593 candidates variants in 1785 index cases (40.4% of total cases, mean of 1.45 per individual) (Fig. 2A), which were distributed to the DITF. After each DITF applied additional prioritisation filters, a total of 678 variants from 566 index cases (31.7% of cases with identified variants; mean of 1.2 variants per individual) were sent to the referring clinical groups for final interpretation (Fig. 2A, Supplementary Table 1). Final interpretation was determined by integrating variant evaluation and patient phenotypic fit. The approach enabled to identify 124 causative variants leading to the diagnosis of 120 RD patients (21.2% of prioritised cases). Among the 124 causative variants identified (Supplementary Table 1), 68 (54.8%) were associated with an autosomal dominant disorder, 44 (35.6%) with an autosomal recessive disorder, 10 (8%) were X-linked, one (0.8%) in mitochondrial DNA and one (0.8%) was a mosaicism. In addition to the 120 diagnosed cases, 26 variants from 25 index cases are still under evaluation (segregation analysis, clinical re-evaluation, SANGER validation, etc.) by the clinical submitting groups (Fig. 2A, C). For an additional 87 index cases, 103 heterozygous variants in phenotype-related candidate genes associated with autosomal recessive disorders were identified. In some of those cases, additional analyses or new data might identify another variant that could finally diagnose the case.

We hypothesised that several cases could have remained undiagnosed when they were originally analysed because knowledge on a specific gene function or variant impact might have been lacking at the time. To further investigate this point, we retrieved, for each of the causative variants, the date when the corresponding gene was first associated with a disease and a pathogenic variant for a specific clinical condition reported in ClinVar (Fig. 2D). In total, 16 (13%) newly identified causative variants were found in genes associated with disorders since 2017 (2 years since data was sent for reanalysis), 11 (9%) between 2015 and 2016, 39 (31%) between 2010 and 2014 and 60 (47%) before 2010. Concerning the clinical significance of the variant, 48 (39%) newly identified causative variants were submitted as pathogenic for a specific disorder to ClinVar since 2017 (2 years since data was sent for reanalysis), 27 (21%) between 2015 and 2016, 40 (31%) between 2010–2014 and 11 (9%) before 2010.

Among the 26 homozygous causative variants (Supplementary Table 2), 15 were identified in experimentally determined consanguineous probands according to ref. [19], being 13 of them within a homozygous stretch of more than 1 Mb (Supplementary Table 2). In order to discard possible false homozygous calls due to a hypothetic heterozygous deletion of the region covering the causative variant in non-consanguineous probands, we cross-checked CNV results provided by the Solve-RD DATF. No deletions in the region of interest were detected.

## Discussion

Constant improvement of bioinformatics methods and advances in genomic understanding to identify and interpret variants highlight the need to periodically re-evaluate unsolved exome/genome cases as stressed by the ACMG [12]. However, to date, the benefit of identifying a new diagnosis in clinical environments is hardly unbalanced compared with the efforts required for re-evaluation. In this study, we present a rapid, scalable and cost-effective approach to programmatically (re)analyse thousands of structured genome-phenome RD-REAL datasets from undiagnosed cases collated as part of the Solve-RD project [13].

We have set up a programmatic system based on a python package to query structured genome-phenome data from the RD-Connect GPAP through its dedicated API. Only sample IDs and filtering parameters need to be defined in the system before attempting a new (re)analysis. Then, the fully automated approach enables to intelligently and flexibly filter genomic data based on clinical, familial, biological and genomic quality information in a rapid (30 s per experiment on average) and massive way (currently >4400 samples tested). The big-data technologies upon which the RD-Connect GPAP is built enable systems to grow by adding more resources as needed. The described approach will allow for the (re)analysis of all the 19,000 exome/genome datasets that Solve-RD aims to collect and the new data it is producing [13].

Despite the use of cutting-edge technologies, and that experts are able to re-evaluate hundreds of cases with the key information at sight, clinical interpretation remains a manual process. In order to facilitate and reduce interpretation efforts, the programmatic output is provided in a meaningful way, integrating relevant genomic, biological and clinical information for referring clinicians and clinical scientists to perform this final step. Results can be enriched with additional annotations and can also include the link to the specific query in the RD-Connect GPAP, enabling the users to explore the variant within a graphical user-interface. We tested the approach with the 4411 affected cases from the first Solve-RD data freeze. All those cases were well characterised and had an exome/genome that had been thoroughly analysed without success. Only the first “low-hanging fruit” filtering approach for rare known pathogenic variants (according to ClinVar) in known disease-causing genes already allowed us to solve 120 undiagnosed index cases (21.2% of prioritised cases). The approach included the use of dedicated ERN associated gene lists to focus on diseases under investigation and limiting the risks of secondary findings. Heterozygous potential candidate variants for autosomal recessive disorders were also identified in 15.3% of the prioritised cases.

The overall positive results obtained from the prioritised variants of this “low-hanging fruit” reanalysis approach can be attributed to several factors. The original exome/genome data reanalysed in this study were sequenced by different centers at different times. This means that the original analyses (including mapping, variant calling, annotation and filtering) were performed with a variety of different tools and databases, likely using different versions and parameters. In addition, the human genome reference used might have been different even if with small changes (e.g. with or without viral and/or decoy sequences). Therefore, the pipeline used in Solve-RD will be in almost all cases somewhat different than the one used in the original analysis, which might have had an effect in unveiling previously undetected variants (e.g ref. [23]). Furthermore, scientific knowledge improves with time, enabling to identify previously undetected associations. In our study, 13% of the newly identified causative variants were in genes not associated with disease in the 2 years prior to reanalysis (described since 2017) and 39% were variants not reported as (likely) pathogenic for similar clinical manifestations at that time. If we assume reanalysis was not performed in the previous 4 years prior to submission, these values increase up to 22% for new disease-causing genes and 60% for newly reported pathogenic variants (e.g. ref. [24] and ref. [25]). Finally, standardised clinical information using HPO, ORDO and OMIM combined with different filtering approaches helped prioritise causative variants in atypical phenotypes (e.g. ref. [25]). This result is aligned with previous studies in the RD-Connect GPAP on a cohort of patients with rare neuromuscular disorders reporting the importance of deep and accurate phenotyping for variant prioritisation [26]. For cases remaining undiagnosed, it might be useful to keep updating the patient phenotypic descriptions with new observations, as this might help identify additional candidate pathogenic variants for the disease and increase specificity of the filtering step, thus lowering the time necessary for variant re-evaluation. In this sense, the RD-Connect GPAP facilitates updating the patient records through its phenotypic module. Remarkably, the interpretation of several causative variants identified in complex genes or regions was possible thanks to the multidisciplinary team of RD experts involved (e.g. ref. [24]).

This first “low-hanging fruit” automated approach managed to solve 2.7% of all clinically heterogeneous undiagnosed and previously negative-exome/genome cases in <37 h of computational time. The flexibility of the system described herein is now being applied to additional strategic reanalyses, varying parameters stringencies and contributing to increase the diagnostic yield. New approaches will focus on the identification of mtDNA variants using specific variant callers [27] or the inclusion of additional clinical resources such as HGMD [8] or Varsome [28]. Indeed, the GPAP already provides direct links to those clinical databases to facilitate variant interpretation and another re-evaluation approach relying on the HGMD database is planned for filtering by (likely) pathogenic variants based on the data available by the user’s license. Several other Solve-RD working groups, focused on the identification of other types of variants or analysis strategies (e.g. copy number variants, repeat expansions or de novo analyses) and/or integrating new –omics generated within the project (e.g. RNA-seq, long read WGS) are joining efforts to unravel additional molecular causes underlying RDs [13].

In comparison and similarly to other iterative reanalysis strategies [10, 29], our approach has three main advantages and time-saving points for clinicians and clinical scientists. First, experts do not need to re-annotate and filter manually with different strategies thousands of cases. Second, they only need to re-evaluate the cases for which at least one candidate variants has been proposed (40.4% of cases in our study). Third, the output file contains all the cases with candidate variants identified and includes key information for their preliminary evaluation.

This method could be adapted to any diagnostic (re)analysis workflow and extended to the whole RD-Connect dataset (currently >13,000 samples) or any subset of interest. Data can be periodically re-evaluated with no additional cost and according to any predefined period of time (e.g. every 6 months or once a year) or after relevant method improvements or database updates. This strategy reduces reanalysis costs and experts’ time-consuming efforts while offering a solution to three out of the four key elements to reinterpret genetic data recently raised by ref. [30]: data storage and re-access, initiation of routine reinterpretation and reinterpretation with novel information.

In summary, we have developed a scalable, cost-effective programmatic approach to drastically decrease turnaround time and effort for periodic data reanalysis. We have illustrated the usefulness of the system by revealing the molecular bases of 120 previously undiagnosed patients with RDs within Solve-RD. This methodology can be implemented systematically in a clinical diagnostic setting for periodic case-level data re-evaluation, as recommended by the ACMG [12].

## Supplementary information

Supplementary_Table_1

Supplementary_Table_2

Solve-RD WG and DITF consortium

Solve-RD consortium
