# Supplementary material for: Solving patients with rare diseases through programmatic reanalysis of genome-phenome data
Source: Eur J Hum Genet. 2021 Jun 1;29(9):1337–47. doi: 10.1038/s41431-021-00852-7 (PMC8440686; doi:10.1038/s41431-021-00852-7)
Supplement: Supplementary file 3 — Solve-RD WG and DITF consortium [file 41431_2021_852_MOESM3_ESM.docx]

**Solve-RD WG and DITF consortium**

**Solve-RD SNV-indel Working group (WG) members and their affiliations.**

* indicates DITF leads.

Enzo Cohen^1^, Isabel Cuesta^2^, Daniel Danis^3^, Anne-Sophie Denommé-Pichon^4,5,6^, Yannis Duffourd^4,6^, Christian Gilissen^7,8^, Mridul Johari^9^, Steven Laurie^10^, Shuang Li^11^, Leslie Matalonga*^10^, Isabelle Nelson^1^, Sophia Peters^12^, Ida Paramonov^10^, Sivakumar Prasanth^13^, Peter Robinson^3^, Karolis Sablauskas^7,8^, Marco Savarese^9^, Wouter Steyaert^7,8^, Ana Töpf^14^, Joeri K. van der Velde^11^, and Antonio Vitobello^4^

Affiliations

^1^Sorbonne Université, INSERM UMRS_974, Center of Research in Myology, 75013 Paris, France.

^2^Institute of Rare Diseases Research, Spanish Undiagnosed Rare Diseases Cases Program (SpainUDP) & Undiagnosed Diseases Network International (UDNI), Instituto de Salud Carlos III, Madrid, Spain.

^22^Ospedale Pediatrico Bambino Gesù, Rome, Italy.

^3^Jackson Laboratory for Genomic Medicine, Farmington, CT 06032, USA.

^4^Inserm - University of Burgundy-Franche Comté, UMR1231 GAD, Dijon, France.

^5^Dijon University Hospital, Genetics Department, Dijon, France

^6^Dijon University Hospital, FHU-TRANSLAD, Dijon, France.

^7^Department of Human Genetics, Radboud University Medical Center, Nijmegen, The Netherlands.

^8^Radboud Institute for Molecular Life Sciences, Nijmegen, the Netherlands.

^9^Folkhälsan Research Center, University of Helsinki, Finland

^10^CNAG‐CRG, Centre for Genomic Regulation (CRG), The Barcelona Institute of Science and Technology, Baldiri Reixac 4, Barcelona 08028, Spain.

^11^Department of Genetics, Genomics Coordination Center, University Medical Center Groningen, University of Groningen, Groningen, The Netherlands.

^12^Institute of Human Genetics, University of Bonn, Bonn, Germany.

^13^MRC Centre for Neuromuscular Diseases and National Hospital for Neurology and Neurosurgery, UCL Queen Square Institute of Neurology, London, UK.

^14^John Walton Muscular Dystrophy Research Centre, Translational and Clinical Research Institute, Newcastle University and Newcastle Hospitals NHS Foundation Trust, Newcastle upon Tyne, UK.

**ITHACA Data Interpretation Task Force (DITF) members and their affiliations.**

* indicates DITF leads.

Siddharth Banka^7,8^, Elisa Benetti^9^, Giorgio Casari^10,11^, Andrea Ciolfi^12^, Jill Clayton-Smith^7,8^, Bruno Dallapiccola^12^, Elke de Boer^4,13^, Anne-Sophie Denommé-Pichon^1,2,26^, Kornelia Ellwanger^14,15^, Laurence Faivre^1,16^, Christian Gilissen^4,5^, Holm Graessner^14,15^, Tobias B. Haack^14^, Anna Hammarsjö^17^, Marketa Havlovicova^18^, Alexander Hoischen^4,8,19^, Anne Hugon^20^, Adam Jackson^16^, Tjitske Kleefstra^4,13^, Anna Lindstrand^17^, Estrella López-Martín^21^, Milan Macek Jr.^18^, Leslie Matalonga^6^, Manuela Morleo^11^, Vicenzo Nigro^11^, Ann Nordgren^17^, Maria Pettersson^17^, Michele Pinelli^11^, Simone Pizzi^12^, Manuel Posada^21^, Francesca Clementina Radio^22^, Alessandra Renieri^9,23,24^, Caroline Rooryck^25^, Lukas Ryba^18^, Martin Schwarz^18^, Marco Tartaglia^12^, Christel Thauvin^1,16^, Annalaura Torella^10,11^, Aurélien Trimouille^26^, Alain Verloes^20,27^, Lisenka Vissers*^4,13^, Antonio Vitobello^1^, Pavel Votypka^18^, Klea Vyshka^20,27^ and Birte Zurek^14,15^

**Affiliations**

^1^Inserm - University of Burgundy-Franche Comté, UMR1231 GAD, Dijon, France.

^2^Dijon University Hospital, Genetics Department, Dijon, France

^3^Dijon University Hospital, FHU-TRANSLAD, Dijon, France.

^4^Department of Human Genetics, Radboud University Medical Center, Nijmegen, The Netherlands.

^5^Radboud Institute for Molecular Life Sciences, Nijmegen, the Netherlands.

^6^CNAG‐CRG, Centre for Genomic Regulation (CRG), The Barcelona Institute of Science and Technology, Baldiri Reixac 4, Barcelona 08028, Spain.

^7^Manchester Centre for Genomic Medicine, St Mary's Hospital, Manchester University Hospitals NHS Foundation Trust, Health Innovation Manchester, Manchester M13 9WL, UK.

^8^Manchester Centre for Genomic Medicine, Division of Evolution and Genomic Sciences, School of Biological Sciences, Faculty of Biology, Medicine and Health, University of Manchester, Manchester, UK.

^9^Med Biotech Hub and Competence Center, Department of Medical Biotechnologies, University of Siena, Italy.

^10^Dipartimento di Medicina di Precisione, Università degli Studi della Campania "Luigi Vanvitelli," Napoli, Italy.

^11^Telethon Institute of Genetics and Medicine, Pozzuoli, Italy.

^12^Genetics and Rare Diseases Research Division, Ospedale Pediatrico Bambino Gesù, IRCCS, 00146 Rome, Italy.

^13^Donders Institute for Brain, Cognition and Behaviour, Radboud University Medical Center, Nijmegen, The Netherlands.

^14^Institute of Medical Genetics and Applied Genomics, University of Tübingen, Tübingen, Germany.

^15^Centre for Rare Diseases, University of Tübingen, Tübingen, Germany.

^16^Dijon University Hospital, Genetics Department and Centres of Reference for Development disorders and intellectual disabilities, FHU TRANSLAD and GIMI InstituteDijon, France.

^17^Karolinska Institutet, Solna, Sweden.

^18^Department of Biology and Medical Genetics, Charles University Prague-2nd Faculty of Medicine and University Hospital Motol, Prague, Czech Republic.

^19^Department of Internal Medicine and Radboud Center for Infectious Diseases (RCI), Radboud University Medical Center, Nijmegen, The Netherlands.

^20^Dept of Genetics, Assistance Publique-Hôpitaux de Paris - Université de Paris, Robert DEBRE University Hospital, 48 bd SERURIER, Paris, France.

^21^Institute of Rare Diseases Research, Spanish Undiagnosed Rare Diseases Cases Program (SpainUDP) & Undiagnosed Diseases Network International (UDNI), Instituto de Salud Carlos III, Madrid, Spain.

^22^Ospedale Pediatrico Bambino Gesù, Rome, Italy.

^23^Medical Genetics, University of Siena, Italy.

^24^Genetica Medica, Azienda Ospedaliero-Universitaria Senese, Italy.

^25^University Bordeaux, MRGM INSERM U1211, CHU de Bordeaux, Service de Génétique Médicale , F-33000 Bordeaux, France.

^26^Laboratoire de Génétique Moléculaire, Service de Génétique Médicale, CHU Bordeaux – Hôpital Pellegrin, Place Amélie Raba Léon, 33076 Bordeaux Cedex.

^27^INSERM UMR 1141 "NeuroDiderot", Hôpital R DEBRE, Paris, France.

**euro-NMD Data Interpretation Task Force (DITF) members and their affiliations.**

Ana Töpf^1^*, Jonathan Baets^2,3,4^, Danique Beijer^2,3^, Gisèle Bonne^5^, Enzo Cohen^5^, Judith Cossins^6^, Teresinha Evangelista^5^, Alessandra Ferlini^7^, Peter Hackman^8^ , Michael G Hanna^9^, Rita Horvath^10^, Henry Houlden^9^, Mridul Johari^8^, Jarred Lau^11^, Hanns Lochmüller^11,12,13,14,15^, William L Macken^9^, Francesco Musacchia^16,17^, Andres Nascimento^18^, Daniel Natera-de Benito^18^, Vincenzo Nigro^16,17^, Giulio Piluso^16^, Veronica Pini^19^, Robert DS Pitceathly^9^, Kiran Polavarapu^11,15^, Pedro M Rodriguez Cruz^6,20^, Anna Sarkozy^19^, Marco Savarese^8^, Rita Selvatici^7^, Rachel Thompson^11^, Annalaura Torella^16,17^, Bjarne Udd^8^, Liedewei Van de Vondel^3,4^, Jana Vandrovcova^9^ and Irina Zaharieva^19^.

^1^John Walton Muscular Dystrophy Research Centre, Translational and Clinical Research Institute, Newcastle University and Newcastle Hospitals NHS Foundation Trust, Newcastle upon Tyne, UK.

^2^Translational Neurosciences, Faculty of Medicine and Health Sciences, UAntwerpen, Antwerp, Belgium

^3^Laboratory of Neuromuscular Pathology, Institute Born-Bunge, University of Antwerp, Antwerpen, Belgium

^4^Neuromuscular Reference Centre, Department of Neurology, Antwerp University Hospital, Antwerpen, Belgium

^5^Sorbonne Université, INSERM, Center of Research in Myology, Paris, France.

^6^Neuromuscular Disorders Group, NDCN, Weatherall Institute of Molecular Medicine, John Radcliffe Hospital, Oxford, UK.

^7^Unit of Medical Genetics, Department of Medical Sciences, University of Ferrara, Italy.

^8^Folkhälsan Research Center, University of Helsinki and Tampere Neuromuscular Center, Finland.

^9^Department of Neuromuscular Diseases, UCL Queen Square Institute of Neurology and The National Hospital for Neurology and Neurosurgery, London, UK.

^10^University of Cambridge, England, UK.

^11^Children's Hospital of Eastern Ontario Research Institute, Ottawa, Canada.

^12^CNAG‐CRG, Centre for Genomic Regulation (CRG), The Barcelona Institute of Science and Technology, Spain.

^13^Division of Neurology, Department of Medicine, The Ottawa Hospital, Ottawa, Canada.

^14^Brain and Mind Research Institute, University of Ottawa, Ottawa, Canada.

^15^Department of Neuropediatrics and Muscle Disorders, Medical Center – University of Freiburg, Faculty of Medicine, Freiburg, Germany

^16^Dipartimento di Medicina di Precisione, Università degli Studi della Campania "Luigi Vanvitelli," Napoli, Italy.

^17^Telethon Institute of Genetics and Medicine, Pozzuoli, Italy.

^18^Neuromuscular Unit, Neuropaediatrics Department, Institut de Recerca Pediàtrica Hospital Sant Joan de Déu, CIBERER, Barcelona, Spain.

^19^Dubowitz Neuromuscular Centre, UCL Great Ormond Street Hospital, London, UK.

^20^Nuffield Department of Clinical Neurosciences, University of Oxford, UK.

**RND Data Interpretation Task Force (DITF) members and their affiliations.**

* indicates DITF leads.

Jonathan Baets^1,2,3^, Peter Balicza^4^, Patrick Chinnery^5^, Alexandra Dürr^6,7,8^, Tobias Haack^9^, Holger Hengel^10,11^, Rita Horvath^12^, Henry Houlden13, Erik-Jan Kamsteeg^14^, Christoph Kamsteeg^14^, Katja Lohmann^15^, Alfons Macaya^16^, Anna Marcé-Grau^16^, Ales Maver^17^, Judit Molnar^4^, Alexander Münchau^15^, Borut Peterlin^17^, Olaf Riess^9,18^, Ludger Schöls^10,11^, Rebecca Schüle-Freyer*^10,11^, Giovanni Stevanin^6,7,8,19,20^, Matthis Synofzik*^10,11^, Vincent Timmerman^21,22^, Bart van de Warrenburg^23^, Nienke van Os^23,24^, Jana Vandrovcova^13^, Melanie Wayand^10,11^ and Carlo Wilke^10,11^

**Affiliations**

^1^Peripheral Neuropathy Research Group, University of Antwerp, Antwerp, Belgium.

^2^Neuromuscular Reference Centre, Department of Neurology, Antwerp University Hospital, Antwerpen, Belgium.

^3^Laboratory of Neuromuscular Pathology, Institute Born-Bunge, University of Antwerp, Antwerpen, Belgium.

^4^Semelweis University Budapest, Hungary

^5^Center for Hereditary Tumor Syndromes, University Hospital Bonn, Bonn, Germany.

^6^Institut National de la Santé et de la Recherche Medicale (INSERM) U1127, Paris, France.

^7^Centre National de la Recherche Scientifique, Unité Mixte de Recherche (UMR) 7225, Paris, France.

^8^Unité Mixte de Recherche en Santé 1127, Université Pierre et Marie Curie (Paris 06), Sorbonne Universités, Paris, France.

^9^Institute of Medical Genetics and Applied Genomics, University of Tübingen, Tübingen, Germany.

^10^Department of Neurodegeneration, Hertie Institute for Clinical Brain Research (HIH), University of Tübingen, Tübingen, Germany.

^11^German Center for Neurodegenerative Diseases (DZNE), Tübingen, Germany.

^12^ University of Cambridge, England, United Kingdom

^13^ Department of Neuromuscular Diseases, UCL Queen Square Institute of Neurology and The National Hospital for Neurology and Neurosurgery, London, UK.

^14^Department of Human Genetics, Radboud University Medical Center, Nijmegen, The Netherlands.

^15^University of Lübeck, Lübeck, Germany

^16^Hospital Vall d’Hebron, Barcelona, Spain

^17^University of Ljubljana, Slovenia

^18^Centre for Rare Diseases, University of Tübingen, Tübingen, Germany.

^19^Institut du Cerveau -ICM, Paris, France.

^20^Ecole Pratique des Hautes Etudes, Paris Sciences et Lettres Research University, Paris, France.

^21^Peripheral Neuropathy Research Group, Department of Biomedical Sciences, University of Antwerp, Antwerp, Belgium.

^22^Institute Born Bunge, Antwerp, Belgium.

^23^Donders Institute for Brain, Cognition and Behaviour, Radboud University Medical Center, Nijmegen, The Netherlands.

^24^Department of Neurology, Radboud University Medical Center, Nijmegen, The Netherlands.

**GENTURIS Data Interpretation Task Force (DITF) members and their affiliations.**

* indicates DITF leads.

Stefan Aretz^1,2^ , Gabriel Capella^3,4^, Richarda M. de Voer*^3,4^, Gareth Evans^5^ , Jose Garcia Pelaez^6,7^, Elke Holinski-Feder^8^, Nicoline Hoogerbrugge^3,4^, Andreas Laner^8^, Carla Oliveira^6,7,9^, Andreas Rump^10^, Evelin Schröck^10^, Anna Katharina Sommer^11^, Verena Steinke-Lange^8^, Iris te Paske^3,4^, Marc Tischkowitz^12^ and Laura Valle^13^

**Affiliations**

^1^Institute of Human Genetics, University of Bonn, Bonn, Germany.

^2^Center for Hereditary Tumor Syndromes, University Hospital Bonn, Bonn, Germany.

^3^Department of Human Genetics, Radboud University Medical Center, Nijmegen, The Netherlands.

^4^Radboud Institute for Molecular Life Sciences, Nijmegen, the Netherlands.

^5^Division of Evolution and Genomic Sciences, School of Biological Sciences, Faculty of Biology, Medicine and Health, University of Manchester, Manchester M13 9WL, UK.

^6^i3S - Instituto de Investigação e Inovação em Saúde, Universidade do Porto, Portugal.

^7^IPATIMUP - Institute of Molecular Pathology and Immunology of the University of Porto, Portugal.

^8^University of Munich, Munich, Germany

^9^Departament of Pathology, Faculty of Medicine, University of Porto, Portugal.

^10^Universty of Dresden, Dresden, Germany

^11^Institute of Human Genetics, University of Bonn, Bonn, Germany.

^12^Department of Medical Genetics, National Institute for Health Research Cambridge Biomedical Research Centre, University of Cambridge, Cambridge, UK

^13^ Instituto de Investigación de Bellvitge, Hospitalet de Llobregat, Spain
